# On the Treatment of Diabetes

**Published:** 1898-08-06

**Authors:** G. Parker

**Affiliations:** Assistant Physician, Bristol General Hospital


### Medical Progress and Hospital Clinics.

[*The Editor will be glad to receive offers of co-operation and contributions from members of the profession. All letters should be addressed to* The Editor, at the Office, 28 & 29, Southampton Street, Strand, London, W.C.]

### ON THE TREATMENT OF DIABETES.

By G. Parker, M.A., M.D., Assistant Physician, Bristol General Hospital.

We meet with this disease under two main types, the acute and the chronic. The latter, sometimes called glycosuria, is found most often in stout elderly persons, may last many years with mild symptoms, and is sometimes associated with gout. The acute type occurs frequently in younger people, presents severe symptoms, and is very fatal. Still in both forms the patient is in constant danger, for any shock, chill, over fatigue, or intercurrent disease may kill very rapidly. Comfortable conditions of life, freedom from worry, and early careful treatment are all important. Saundby notices that the prognosis is good in cases following after an injury or acute illness, or from gout.

There is little in the appearance of sufferers to lead to a diagnosis, but it is rare in either form to find them anæmic, even when the body is wasted. In a very few instances bronzing of the skin is seen, but generally the face is normal or flushed, and has an anxious expression. The first symptoms may be loss of flesh, cramping pain in the calves, persistent thirst, increased frequency of micturation, and the excretion of large quantities of urine. At other times attention may be drawn to the disease by the appearance of eczema, pruritus, dyspnœa, chapped fingers, cataract, perforating ulcer, or even by the aromatic odour of the patient when coma is imminent, or by vomiting, rapid pulse, the loss of knee jerks, or gangrene. The sugar in the urine is detected most certainly by the fermentation test. Fehling's reaction is also reliable, except when glycuronic acid, salicylates, chloral, or uric acid in excess are present in the urine. The phenyl hydrazin test is the most delicate and a quicker operation than fermentation. When urine cannot be obtained, Williamson's blood test is useful, in which a drop of blood decolourises a weak solution of methylene blue when boiled with it for a few minutes. The sugar present in the blood or urine is usually dextrose or glucose, and is increased in the day urine or after food, especially if this contains sugar or starch. In the mildest form it disappears when no carbohydrates are given, while in the severest cases it is present however rigid a diet we may adopt. The amount often decreases under moderate exercise, and when febrile affections, fatal phthisis, or granular kidney disease come on; but it is increased by worry, shock, long journeys, or careless feeding. Albumen is not infrequently present in small quantities, possibly from the irritation of the saccharine urine, and occasionally typical Bright's disease takes the place of diabetes with rapidly fatal results. Another symptom of bad omen, though it is uncertain how it is caused, is the presence of the Burgundy red reaction given by adding a few drops of ferric chloride solution. It is a fairly good danger signal of impending coma, and should lead us to modify a too rigid diet, and to remedy any existing constipation. Certain drugs, such as salicyn or antipyrin, may, however, cause a nearly similar reaction. Acetone, shown by Legal or Le Nobel's tests, is an even stronger warning of coma, of which other early symptoms are languor, altered breathing, epigastric pain, quickened pulse, an aromatic odour, diminished urine, and perhaps mental excitement. Still, acetone may occur without coma developing, and is found in other diseases.

Now, in treatment we have to guard against several dangers, besides seeking to remove or lessen the excretion of sugar. Above all things, the general health of the patient should be watched, and his weight taken every week. Constipation and the first signs of phthisis should be noted, as well as the amount of sugar, which should be calculated every week by the fermentation test. After the patient has been under our care for some days on ordinary diet, a gradual restriction should be made, and a diet of nitrogen and fat only should be substituted for at least a week. If the sugar has then disappeared entirely we have to do with a mild case only, or there may be a great but not complete reduction of the sugar. Williamson points out that when albumen is present it may be necessary to limit the flesh food also before we can get rid of the sugar. When we have attained the maximum effect from food and drugs, we may try the effect of adding carbohydrates to the diet. Saundby recommends a baked potato taken daily as the first step. If no increase of sugar is found a further advance may be made. The weight of the patient is, after all, one of the chief indications of a suitable diet. If this decreases, and the sugar increases upon any relaxation of the regimen, we must go back. Thus it may be necessary to continue on a strict diet for years, and even then we may fail to get less than 500 grains of sugar in the urine daily. More often some relaxation is desirable after awhile, and even the loss of weight, like the iron reaction, may be sometimes stopped by the addition of carbohydrates. Generally speaking it has been laid down that green vegetables and fatty foods should be given freely, nitrogenous ones in moderation, sugar not at all, milk in moderate quantities because of the lactose contained in it; while carbohydrates, such as starch, should be used to such an extent as is tolerated by the organism. Of animal foods liver and shell-fish must be denied, but bacon, eggs, butter, and fats of all kinds are most useful. Williamson speaks highly of Devonshire cream, and of cream stirred up in a large quantity of water and again skimmed, which yields a sugar-free cream to which white of egg may be added. One great difficulty is to find satisfactory substitutes for bread, for they are expensive to buy and many are absolute frauds. Very few samples of gluten bread have as little as 5 per cent. of starch, many containing 25 per cent. and over, while ordinary bread only shows 50 per cent. So that if a patient, instead of eating such gluten bread, only takes half the quantity of ordinary bread, he is just as well off, and enjoys his meal much better. All such bread should be tested by a drop of iodine in potassium iodide solution, which gives a bluish-black colour if much starch is present. So, too, the fermentation test should be used for almond or cocoanut meal. Saundby's advice, that every practitioner should himself test his patient's food by these simple plans, is worthy of remembrance. Cooks, too, are apt to add flour or sugar to sauces, and in dressing meat, and usually find great difficulty in baking gluten or cocoanut meal. Here we may notice that good cocoanut meal, the cheapest of all substitutes for flour, can be deprived of the little sugar it contains by adding some yeast with water, and keeping it in a warm place for half an hour or longer; while almond flour may be freed from its sugar by placing it in a linen bag in a pan of boiling water for fifteen minutes, to which a few drops of vinegar have been added (Seegen). Aleuronat flour can be obtained with only 7 per cent. of starch, and Williamson strongly recommends cakes or biscuits made by baking equal parts of cocoanut meal and aleuronat flour with an egg and a little salt. Yeast is first added, and then the small cakes of dough are baked in a quick oven for 20 or 30 minutes. The cost of cocoanut meal is, he remarks, only 4½d. a pound, while aleuronat flour can be obtained from Westphalia at 9½d. This gives a good and palatable bread at much less than the cost of ordinary gluten, which is far beyond the means of poor patients.

Whatever bread substitutes we employ, they are to be used either temporarily for distinguishing the exact type of the disease; or for removing complications such as gangrene; or for entirely getting rid of the whole of the sugar in mild cases; or finally, for longer periods in more severe cases, if they are found to cause a great reduction of sugar and improve the general state. Some patients, indeed, do best on them for years, but if their use is followed by symptoms of impending coma or a great loss of weight, recourse must be had to ordinary bread in small quantities, or to potatoes, either baked or in the form of chips which have been fried in oil. Toast seems to have no advantages over ordinary bread, but there are useful varieties of Soya, bran, almond, and other biscuits to be used for a change, if only you will take the trouble to test each sample for yourself. The question has been asked what diet shall we give when nephritis is present, as well as diabetes, and a meat diet is clearly dangerous. Each case must be decided to some extent on its merits; but it is best to reduce the meat as much as possible, giving fats and such carbohydrates as the diabetes will allow.

As to drink, no attempt now is made to limit strictly the fluid imbibed. Water, tea, coffee, very dry wines, spirits and water, tartrate of potash, lemonade sweetened with saccharine, water acidulated with phosphoric acid, and similar fluids may be administered to relieve the thirst.

With regard to drugs, a large number have been tried, but the reliable ones are very few indeed. Still, as we cannot in every case be sure of the cause of the diabetes, whether it be gouty, pancreatic or otherwise, and also since from unexplained idiosyncrasies individuals have improved under the use of various remedies, such as the salicylates, we may often make trial of one or other of them. However, none are so certain as opium and its derivatives, morphine and codeia; and generally, as soon as we have reduced the sugar as much as possible by rest and diet, one of these should be given. We may commence with half-grain doses of opium, or one grain of codeia three times a day, if neither coma or Bright's disease is to be feared, and the quantity may be increased to three times that amount or even more, with good results in the reduction of sugar of the urine excreted. Of course, measures must be taken to prevent the constipating action of opium, and elderly patients should be warned not to be alarmed at the disturbing dreams which it is apt to produce for the first night or two.

West has found good results from uranium nitrate gradually increased, and if we had good means of diagnosing pancreatic diabetes it might be possible to graft portions of the pancreas under the skin with success. For coma little can be done beyond purgation, and the freest administration of alkalies by the mouth and by injection; but the scrupulous care in treatment, which I have endeavoured to emphasise, should go far to prevent its occurrence.

